# Microbiome-metabolomics signature in anorexia nervosa (AN) before and after weight regain

**DOI:** 10.1192/j.eurpsy.2021.943

**Published:** 2021-08-13

**Authors:** F. Marciello, A.M. Monteleone, J. Troisi, G. Cascino, G. Serena, R. Dalle Grave, P. Monteleone

**Affiliations:** 1 Department Of Medicine, Surgery And Dentistry, University of Salerno, Baronissi/Salerno, Italy; 2 Department Of Psychiatry, University of Campania “Luigi Vanvitelli”, Naples, Italy; 3 (ebris), European Biomedical Research Institute of Salerno, Salerno, Italy; 4 Mucosal Immunology And Biology Research Center, Massachusetts General Hospital-Harvard Medical School, Boston, United States of America; 5 Department Of Eating And Weight Disorders, Villa Garda Hospital, Garda/Verona, Italy; 6 Department Of Medicine, Surgery And Dentistry, University of Salerno, Baronissi/Salerno, Italy

**Keywords:** anorexia nervosa, Gut Microbiota, eating disorders, Metabolomic

## Abstract

**Introduction:**

The intestinal microbiota has been indicated to have a role in the pathophysiology of AN.

**Objectives:**

Aim of this study was to analyze fecal microbiome profiles of AN women before and after weight restoration and to combine them with fecal metabolomic profiles according to a multi-omics approach.

**Methods:**

The gut microbiome and fecal metabolites were characterized in 21 underweight AN women and after weight restoration and compared with those of 20 healthy women. Microbiome data were correlated with the relevant fecal metabolites.

**Results:**

AN subjects showed a decreased intra-individual bacterial richness, an increased Bacteroidetes-to-Firmicutes abundance ratio and significant changes in the relative abundances of several bacteria at different order levels in both the underweight and weight-restored condition compared to healthy women. The untargeted metabolomic procedure allowed the characterization of 224 metabolites involved in energy, lipid and amino acid metabolism. A genetic algorithm identified 49 relevant metabolites. The relationships among these fecal metabolites and bacteria genera showed structures of different complexity in the 3 groups. In particular, a quarter of those relationships showed a divergent direction in the acute phase of AN than in the weight-restored phase or normal controls. Finally, in acute AN 70% of those correlations showed a negative sign suggesting a prevalent metabolites consummation by gut microbiome.

**Conclusions:**

Our results provide a picture of the connections between gut bacteria and fecal metabolites in both the acute phase of AN and after short-term weight restoration. Further studies should aim to investigate the significance of gut microbiome perturbations in development and treatment of AN.

